# A General Formula for Fan-Beam Lambda Tomography

**DOI:** 10.1155/2007/95295

**Published:** 2007-08-27

**Authors:** Hengyong Yu, Ge Wang

**Affiliations:** Biomedical Imaging Division, VT-WFU School of Biomedical Engineering and Science, Virginia Tech, Blacksburg, VA 24061, USA

In [1], we proved that a lambda tomography (LT) image can be reconstructed based on either an even or an odd data extension. Unfortunately, the proof related to the odd data extension is not generally correct, because equation (31) is a necessary condition instead of a sufficient one for equation (32). That is, in the general case, equation (32) cannot be obtained from equation (31). However, the reconstruction formula without any data extension remains correct in practical applications where the object support is constrained in a convex region surrounded by a scanning trajectory. We apologize for any confusion that [1] might have caused. For more details, please refer to our new paper [2].
